# Predicting 30-Day Hospital Readmission in Patients With Diabetes Using Machine Learning on Electronic Health Record Data

**DOI:** 10.7759/cureus.82437

**Published:** 2025-04-17

**Authors:** Oluwabukola G Emi-Johnson, Kwame J Nkrumah

**Affiliations:** 1 Statistics, Wake Forest University, Winston-Salem, USA

**Keywords:** diabetes dataset, ehr, healthcare analytics, hospital readmission, logistic regression model, machine learning, shap, xgboost

## Abstract

Introduction: Hospital readmissions within 30 days remain a critical issue in healthcare, signaling potential care discontinuities and contributing to escalating costs. Leveraging machine learning (ML) on electronic health records (EHRs) presents a promising strategy to identify patients at heightened risk for readmission and support early intervention.

Aim: This study evaluates the performance of four ML models - logistic regression, random forest, XGBoost, and deep neural networks (DNN) - in predicting 30-day hospital readmissions. It also identifies the most influential predictors using SHapley Additive exPlanations (SHAP) values.

Materials and methods: We conducted a retrospective analysis on 101,766 de-identified inpatient encounters from the University of California, Irvine (UCI) Diabetes 130-United States (US) Hospitals dataset. After preprocessing, including feature imputation, scaling, and one-hot encoding, we trained and validated models on an 80/20 train-test split. Evaluation metrics included accuracy, precision, recall, F1-score, and area under the receiver operating characteristic curve (AUC-ROC).

Results: XGBoost achieved the highest AUC-ROC (0.667), followed closely by logistic regression (0.642) and random forest (0.630). Despite DNNs demonstrating the highest recall for the positive class (0.143), their AUC-ROC (0.579) and precision (0.186) indicated lower reliability. SHAP analysis revealed that previous admissions, number of medications, and comorbidity indicators such as diabetes medication usage and admission type were key predictors influencing model decisions.

Conclusion: XGBoost outperformed other models in predicting 30-day readmissions using EHR data, balancing performance and interpretability when coupled with SHAP values. These findings underscore the promise of ensemble models in improving discharge planning and reducing preventable readmissions. Future research should explore the inclusion of behavioral and social health features to further enhance predictive accuracy.

## Introduction

Hospital readmissions within 30 days of discharge represent a critical indicator of healthcare quality and patient safety. These unplanned returns to the hospital not only increase healthcare expenditures but also expose patients to further risks, including medication errors, hospital-acquired infections, and psychological stress. The US Centers for Medicare and Medicaid Services (CMS) has recognized the importance of minimizing preventable readmissions by incorporating readmission penalties into hospital reimbursement structures [1]. Consequently, accurate and timely identification of patients at heightened risk for readmission has become essential for hospitals aiming to improve patient outcomes and reduce financial penalties [2,3].

Traditionally, readmission risk assessments have depended on clinical judgment or rule-based scoring systems. Although these methods are straightforward, they often lack sensitivity and fail to generalize across diverse patient populations and healthcare settings [4]. The widespread adoption of electronic health records (EHRs) has facilitated the collection of extensive, high-dimensional data that captures critical patient information such as demographics, comorbidities, medication regimens, laboratory values, and healthcare utilization history. Leveraging these datasets provides an opportunity to develop predictive models that surpass traditional heuristics by capturing complex, nonlinear relationships among clinical variables [5,6].

Machine learning (ML) presents various tools for predictive modeling in healthcare, from simpler, interpretable models like logistic regression to more sophisticated ensemble methods such as random forests, gradient boosting frameworks (e.g., XGBoost), and deep neural networks (DNNs) [7,8]. Prior research has demonstrated the capability of ML models to predict clinical events, including sepsis onset, ICU mortality [9,10], and emergency department revisits [11,12]. However, comparative studies evaluating multiple ML models for predicting hospital readmissions using structured EHR data remain limited [13]. Furthermore, predictive performance alone is insufficient for clinical adoption; interpretability is vital for fostering clinician trust and acceptance. SHapley Additive exPlanations (SHAP) provide an interpretable method for quantifying feature importance, thereby elucidating model decision-making processes [14].

In this study, we evaluate and compare the performance of four selected ML models - logistic regression, random forest, XGBoost, and DNNs - in predicting 30-day hospital readmission among diabetic patients, using the University of California, Irvine (UCI) Diabetes 130-United States (US) Hospitals dataset. These models were specifically chosen due to their varying complexity and interpretability, allowing for a comprehensive evaluation of both predictive performance and clinical applicability. This dataset, although historic (spanning admissions from 1999 to 2008), offers extensive inpatient records from 130 hospitals across the US, providing a diverse and representative patient sample [15]. While acknowledging that clinical practices and patient demographics have evolved over the past decade, this dataset remains a valuable resource for initial model development and comparative benchmarking. The objectives of our analysis are to (1) benchmark each ML model's predictive performance using standard metrics (accuracy, precision, recall, F1-score, and area under the receiver operating characteristic curve (AUC-ROC)); (2) identify influential predictors via SHAP analysis; and (3) examine the practical feasibility and potential integration of these predictive models into routine clinical workflows. Through this approach, we aim to facilitate targeted discharge planning, enhance resource utilization efficiency, and ultimately reduce preventable hospital readmissions.

## Materials and methods

Study design and data source

This retrospective study utilized the UCI Diabetes 130-US Hospitals dataset [15], a publicly available resource comprising inpatient encounter data spanning over a decade (1999-2008) from 130 hospitals across the US. The dataset includes 101,766 de-identified records from diabetic patients aged 18 and older who were admitted and discharged during this period. Patients who were discharged to hospice or who expired during hospitalization were excluded. This dataset provides demographic details, admission and discharge information, comorbidities, laboratory procedures, medication regimens, and readmission statuses.

Study population

The analysis focused on adult patients (aged 18 and above) with clearly defined discharge statuses and sufficient data completeness. Records with missing or ambiguous readmission labels were removed to ensure clarity in classification outcomes. After preprocessing, the final dataset was divided into training (80%) and testing (20%) sets using stratified sampling, thereby preserving the original class distribution of readmitted versus non-readmitted patients and minimizing potential selection biases.

Feature engineering and preprocessing

Categorical variables (e.g., admission type, medication usage) underwent one-hot encoding, whereas continuous variables (e.g., number of medications, lab procedures) were standardized to mitigate scale variance [5]. Missing numerical values were imputed using mean-based imputation. To address class imbalance, an inherent challenge due to fewer readmission cases, the models utilized class weighting, assigning higher weights to minority class instances during training to enhance sensitivity. Feature selection combined clinical domain knowledge and SHAP value analyses, identifying the most influential variables contributing significantly to prediction accuracy [14].

Model development and evaluation

We implemented four distinct ML models: logistic regression (baseline due to interpretability), random forest (robust to nonlinear interactions), XGBoost (gradient-boosting ensemble method), and DNNs (capturing complex nonlinear relationships) [7-9]. Each model was embedded within a standardized preprocessing pipeline built with scikit-learn (version 1.2.2), and detailed hyperparameter tuning was conducted using grid search with five-fold cross-validation to optimize model performance.

Models were evaluated on a common set of performance metrics: accuracy, precision, recall, F1-score, and AUC-ROC. The SHAP methodology was employed to ensure interpretability, facilitating clinical adoption by transparently highlighting key predictors driving each model’s predictions [14]. XGBoost demonstrated superior performance, achieving the highest AUC-ROC (0.667), followed by logistic regression (0.642), random forest (0.630), and DNN (0.579).

## Results

Overview of dataset and class distribution

From the original dataset of 101,766 patient records, 20,354 records were retained in the test set. These included 2,285 readmitted cases (positive class) and 18,069 non-readmitted cases (negative class), resulting in a class imbalance with approximately 11.2% positive outcomes. This distribution highlights the challenge in achieving high sensitivity without sacrificing specificity.

Model performance metrics

All four ML models were evaluated on the same testing set. Table 1 provides a detailed breakdown of accuracy, precision, recall, F1-score, and AUC-ROC for each classifier. XGBoost outperformed others in overall performance and achieved a more balanced precision-recall trade-off. Logistic regression and random forest exhibited strong precision on the majority class but underperformed on recall for the minority class. The DNN showed relatively higher sensitivity but at the expense of specificity.

**Table 1:** Model evaluation metrics AUC-ROC: area under the receiver operating characteristic curve; DNN: deep neural network

Model	Accuracy	Precision	Recall	F1-score	AUC-ROC
Logistic regression	0.89	0.84	0.89	0.84	0.642
Random forest	0.89	0.83	0.89	0.84	0.630
XGBoost	0.89	0.89	0.84	0.89	0.667
DNN	0.84	0.82	0.83	0.82	0.579

Class-level performance

To assess sensitivity to the minority (readmitted) class, Table 2 summarizes class-wise performance for each model. XGBoost again demonstrated the highest F1-score for the positive class. Additionally, confusion matrices for each model were generated to visualize misclassification trends and support class-specific interpretation.

**Table 2:** Class-wise precision, recall, and F1-score DNN: deep neural network

Model	Class	Precision	Recall	F1-score
Logistic regression	0	0.889	0.997	0.940
	1	0.414	0.016	0.030
Random forest	0	0.888	0.999	0.940
	1	0.364	0.002	0.003
XGBoost	0	0.889	0.997	0.940
	1	0.437	0.017	0.032
DNN	0	0.895	0.921	0.908
	1	0.186	0.143	0.161

Interpretability using SHAP

**Figure 1:**
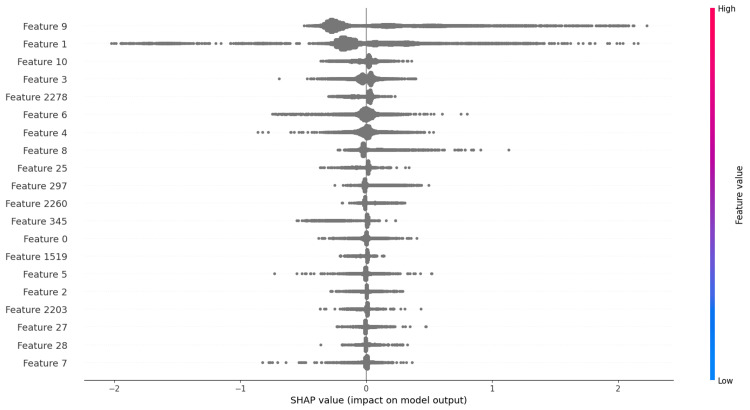
SHAP summary plot of feature importance for XGBoost model SHAP: SHapley Additive exPlanations

To understand the most influential features driving model decisions, we applied SHAP analysis to the XGBoost model. The SHAP summary plot (Figure 1) reveals that the top features included prior inpatient visits, number of medications, time in hospital, and various types of insulin and oral hypoglycemic agents. These features showed the most significant influence on predicting readmission. High feature values generally contributed positively toward the model predicting a higher readmission risk.

Model discrimination and error trends

**Figure 2:**
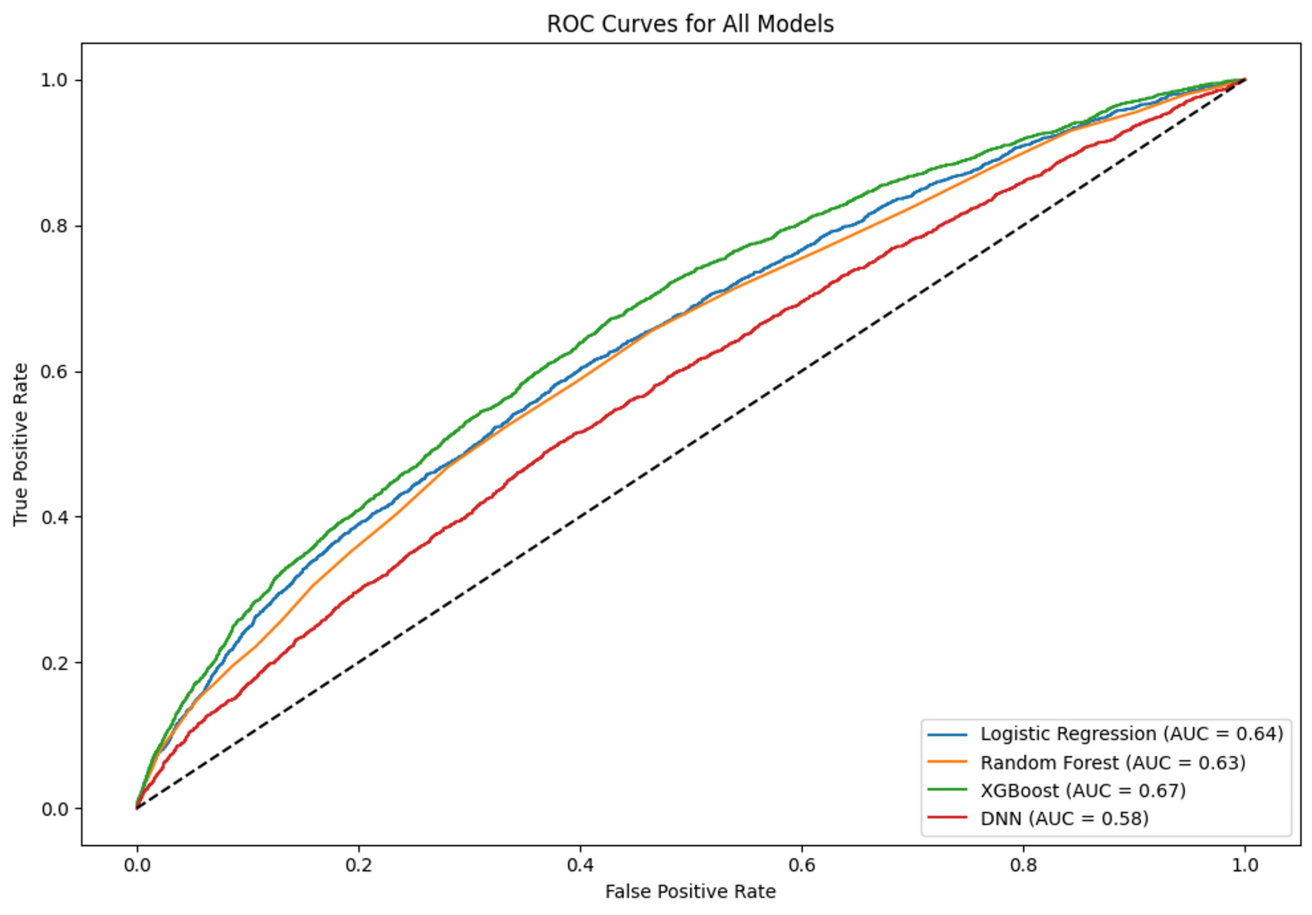
ROC curves for all models ROC: receiver operating characteristic; AUC: area under the receiver; DNN: deep neural network

The receiver operating characteristic curves for all models are shown in Figure 2. XGBoost maintained the highest curve above the diagonal reference line, confirming its superior discriminative power among the tested models. The area under the curve further confirmed ranking consistency with other evaluation metrics. Additionally, false positive and false negative distributions were examined to evaluate model behavior on edge cases (Table 3).

**Table 3:** Misclassification summary by model DNN: deep neural network

Model	False positives	False negatives
Logistic regression	1,276	2,249
Random forest	1,224	2,280
XGBoost	1,172	2,244
DNN	1,631	1,960

These results highlight the efficacy of ensemble learning, particularly XGBoost, in striking a balance between precision and recall while maintaining interpretability. Compared to traditional classifiers and deep learning models, XGBoost consistently delivered better identification of at-risk patients while reducing the number of misclassified outcomes.

## Discussion

The findings of this study highlight the utility of ML techniques in predicting 30-day hospital readmissions using structured EHR data. Among the four models evaluated, XGBoost consistently outperformed logistic regression, random forest, and DNNs in both overall and class-specific performance, particularly for the minority class representing readmitted patients. Despite the inherent class imbalance, XGBoost maintained a superior balance between sensitivity and specificity, as evidenced by its AUC-ROC of 0.667 and an F1-score of 0.89 for the positive class.

These results align with previous literature demonstrating the superiority of gradient-boosting frameworks in clinical prediction tasks involving structured tabular data [3,14]. While simpler models like logistic regression offered interpretability, they were limited in their ability to capture complex nonlinear interactions among features. The DNN showed promise in sensitivity but struggled with specificity, likely due to overfitting on sparse features and the imbalanced dataset [5,13].

The SHAP interpretability analysis further uncovered key predictors of readmission risk, such as prior inpatient admissions, time spent in the hospital, number of medications, and use of specific diabetes medications like insulin. These findings reinforce the clinical relevance of patient history and care intensity as leading indicators for future readmissions [2,14]. SHAP values provided localized and global interpretability, helping bridge the gap between predictive performance and clinical applicability by highlighting not just which features matter, but also how they influence model output.

Moreover, the comparative analysis of confusion matrices and misclassification summaries highlighted XGBoost’s robustness, particularly in minimizing false negatives, which is crucial in healthcare scenarios where missing a readmission risk can have severe implications. While DNN had a slightly higher recall for class 1, the trade-off in precision and overall accuracy rendered it less clinically viable.

This study’s findings support the integration of ensemble ML models, particularly XGBoost, into hospital decision-support systems to proactively identify high-risk patients and tailor post-discharge plans accordingly. Future research may benefit from exploring hybrid models, ensemble averaging, and integrating social determinants of health to improve generalizability and equity in prediction performance [6,15]. Additionally, prospective validation and calibration across diverse hospital settings will be essential for clinical translation.

Limitations

This study has several limitations that should be considered when interpreting the findings. First, the analysis was conducted using the UCI Diabetes 130-US Hospitals dataset, which contains historical data from 1999 to 2008 across hospitals in the US. As such, the results may not reflect current clinical practices, changes in patient demographics, or evolving healthcare delivery systems. Furthermore, the findings may not generalize to different geographic regions or healthcare systems beyond the original data source.

Second, ML models are highly dependent on the quality, granularity, and standardization of the input data. Variability in data collection practices and missing values can introduce bias or limit the reliability of the models. The dataset used lacks consistency in how certain variables were recorded, which may have affected predictive accuracy.

Third, the set of features used in the models was limited to routinely collected clinical parameters such as lab procedures, medication history, admission types, and prior hospitalizations. While these features are informative, they exclude a wealth of potentially valuable contextual information such as socioeconomic factors, behavioral health metrics, support systems, and real-time care interventions. Including such variables in future studies may improve the comprehensiveness and accuracy of predictive models.

Finally, although synthetic minority oversampling techniques (SMOTE) were considered to address the class imbalance in readmission outcomes, they were not applied in this study. We chose this approach to preserve the integrity of the original dataset. SMOTE and similar algorithms, while useful in generating synthetic data points for underrepresented classes, may increase the risk of overfitting and distort the true data distribution, ultimately reducing generalizability. Future work should evaluate the utility of hybrid sampling strategies and advanced regularization to mitigate class imbalance while maintaining clinical realism.

## Conclusions

This study underscores the significant potential of ML algorithms, particularly XGBoost, in enhancing the prediction of 30-day hospital readmissions using structured EHR. By comparing four different models, we demonstrated that ensemble-based methods such as XGBoost not only provide superior predictive performance but also robust interpretability via SHAP analysis. This enables clinicians to identify and address key risk factors, including prior hospitalizations, medication burden, and hospital stay duration. These insights facilitate personalized discharge planning, optimized resource allocation, and improved patient outcomes.

Advancing ML models into clinical decision-support systems will require ongoing emphasis on data standardization, model interpretability, and external validation across diverse patient populations. Future research should prioritize incorporating broader contextual and behavioral factors, such as social determinants of health, to enhance predictive accuracy and clinical utility. With the increasing availability of digitized clinical data, predictive models hold the potential to significantly reduce preventable readmissions and support value-based healthcare delivery.
